# Bioengineering-tissue strategies to model mammalian implantation *in vitro*

**DOI:** 10.3389/fbioe.2024.1430235

**Published:** 2024-07-26

**Authors:** Georgia Pennarossa, Sharon Arcuri, Agata Zmijewska, Elena Orini, Fulvio Gandolfi, Tiziana A. L. Brevini

**Affiliations:** ^1^ Laboratory of Biomedical Embryology and Tissue Engineering, Department of Veterinary Medicine and Animal Science, Università Degli Studi di Milano, Lodi, Italy; ^2^ Department of Animal Anatomy and Physiology, Faculty of Biology and Biotechnology, University of Warmia and Mazury in Olsztyn, Olsztyn, Poland; ^3^ Department of Agricultural and Environmental Sciences—Production, Landscape, Agroenergy, Università Degli Studi di Milano, Milan, Italy

**Keywords:** 3D endometrial model, trophoblast spheroid, chemical reprogramming, scaffolds, microbioreactors

## Abstract

During mammalian implantation, complex and well-orchestrated interactions between the trophectoderm of implanting blastocysts and the maternal endometrium lead to a successful pregnancy. On the other hand, alteration in endometrium-blastocyst crosstalk often causes implantation failure, pregnancy loss, and complications that result in overall infertility. In domestic animals, this represents one of the major causes of economic losses and the understanding of the processes taking place during the early phases of implantation, in both healthy and pathological conditions, is of great importance, to enhance livestock system efficiency. Here we develop highly predictive and reproducible functional tridimensional (3D) *in vitro* models able to mimic the two main actors that play a key role at this developmental stage: the blastocyst and the endometrium. In particular, we generate a 3D endometrial model by co-culturing primary epithelial and stromal cells, isolated from sow uteri, onto highly porous polystyrene scaffolds. In parallel, we chemically reprogram porcine adult dermal fibroblasts and encapsulate them into micro-bioreactors to create trophoblast (TR) spheroids. Finally, we combine the generated artificial endometrium with the TR spheroids to model mammalian implantation *in vitro* and mimic the embryo-maternal interactions. The protocols here described allow the generation of reproducible and functional 3D models of both the maternal compartment as well as the implanting embryo, able to recreate *in vitro* the architecture and physiology of the two tissues *in vivo*. We suggest that these models can find useful applications to further elucidate early implantation mechanisms and to study the complex interactions between the maternal tissue and the developing embryos.

## 1 Introduction

Impaired reproductive performance and infertility in domestic species represent the major causes of economic losses in the livestock systems, worldwide. In the sow, reduction in farrowing rates, due to pregnancy loss, is the most significant manifestation of this phenomenon. Recent advancements in porcine reproductive physiology understanding have shown a strict correlation among poor oocyte developmental competence, ovarian activity decrement, hormonal level alterations and infertility (Bertoldo et al., 2012; Kemp et al., 2018; Peltoniemi et al., 2019; Pennarossa et al., 2021; 2022; Sirait et al., 2021). On the other hand, little is currently known about the mechanisms leading to pregnancy loss associated with uterine dysfunctions. It is well known that uterine infections and persistent inflammations, as well as thermal stress and environmental exposure to endocrine-disrupting chemicals, can negatively affect mammalian reproduction (Wang et al., 2013; Kim and Kim, 2017; Laws et al., 2021; Taylor et al., 2023). This is particularly impacting during the implantation process, which requires highly complex and properly orchestrated interactions between the maternal tissue and the developing embryos (Mardon et al., 2007; Yoshinaga, 2008; Harduf et al., 2009; Brevini et al., 2012; Wang et al., 2012). At this developmental stage, the two main actors are represented by the conceptus and the endometrium, which plays a key role during this as well as along all the pregnancy period, influencing overall uterine receptivity, early embryo development, hormone production, pregnancy maintenance, and serving as a protection barrier against external agents. Several reports, mostly based on a descriptive approach, demonstrated a strict correlation between abnormal implantation and reproductive failure in the mouse (Horcajadas et al., 2004; 2007; Mirkin et al., 2004; Lea and Sandra, 2007) as well as in primate (Fazleabas et al., 2004; Einspanier et al., 2006). These studies were carried out *in vivo* and are not able to accurately recapitulate and/or mimic early implantation stages in large mammals (Wang et al., 2012; Bashiri et al., 2018; Ma et al., 2022).

Considering the great importance of understanding mammalian endometrial physiology, in both healthy and pathological conditions, and given the practical limitations in obtaining maternal and embryonic/fetal tissues, there is an urgent need of highly predictive tridimensional (3D) *in vitro* models, able to mimic the complex, multicellular architecture and functions of the two main structures involved in the implantation process. Many efforts have been therefore dedicated to establishing advanced *in vitro* tissue models that bridge the gap between the *in vivo* complexity and the over-simplified conventional two-dimension (2D) *in vitro* cultures (Pereira et al., 2023; Junior et al., 2024). At present, 3D scaffolds obtained from natural biomaterials, such as matrigel, collagen and other compounds, have been used to model the endometrial tissue *in vitro* (Alaminos and Campos, 2023). However, these strategies are not able to recreate the functional communications between epithelial and stromal cells (Fitzgerald et al., 2021; Alaminos and Campos, 2023). They also lack reproducibility, due to the use of complex protocols, to batch-to-batch variations and because of the difficulties in epithelial and stromal cell long-term co-culture (Fitzgerald et al., 2021; Rawlings et al., 2021).

In the present study, we develop a reproducible functional 3D endometrial model by co-culturing primary epithelial and stromal cells, isolated from sow uterine tissues, onto commercially available highly porous polystyrene scaffolds that encourage 3D cell aggregation and maintenance of the original phenotype, while supporting long-term growth. We then use a micro-bioreactor-based culture system to create trophoblast (TR) spheroids, that mimic the placental embryo compartment, starting from porcine adult dermal fibroblasts chemically reprogrammed into TR-like cells. Finally, we combine the artificial endometrium and the TR spheroids to model mammalian implantation *in vitro* and simulate embryo-maternal interactions.

## 2 Materials and methods

All reagents were purchased from Thermo Fisher Scientific unless otherwise indicated.

### 2.1 Ethic statement

Porcine cells were obtained from fresh uteri and skin biopsies collected at the local abattoir from adult animals. All experiments were performed in accordance with the approved guidelines.

### 2.2 Creation of 3D endometrial model

#### 2.2.1 Endometrial stromal fibroblast (eSF) and epithelial cell (eEC) isolation, growth, and maintenance onto standard plastic dishes

Three uteri were collected from a local abattoir from five nonpregnant swine under 30 months of age, transported to the laboratory on ice and kept on it while processing. After extensive cleaning, uterine horns were longitudinally excised and opened. The inner surface was dissected with a sterile scalpel to separate mucosa and submucosa endometrial tissues from the muscularis mucosa, and the obtained tissues were cut in small pieces of approximately 2 mm^3^.

For eSF isolation, 5 fragments were plated with submucosa layer facing towards each 0.1% porcine gelatin (Sigma-Aldrich) pre-coated Petri dish (Sarstedt) and cultured in Dulbecco’s modified Eagle’s medium (DMEM), supplemented with 20% Fetal Bovine Serum (FBS), 2 mM glutamine (Sigma-Aldrich) and antibiotics (Sigma-Aldrich). After 6 days of culture, fragments were carefully removed and fibroblasts were maintained in the medium described above supplemented with 10% FBS, grown in 5% CO_2_ at 37°C, and passaged twice a week at 1:3 ratio.

For eEC isolation, 5 fragments were plated with epithelial layer facing towards each 0.1% porcine gelatin (Sigma-Aldrich) pre-coated Petri dish (Sarstedt) and cultured in the medium DMEM/F12, supplemented with 20% FBS, 2 mM glutamine (Sigma-Aldrich) and antibiotics (Sigma-Aldrich). Tissue fragments were then carefully removed and differential plating were carried out. Specifically, cells were detached from culture supports and plated onto new 0.1% porcine gelatin pre-coated Petri dishes and incubated for 1 h. Non-adherent cells in the supernatant fraction were collected and plated onto new dishes. This procedure was repeated at least 4 time, depending on cell culture purity. Once isolated, eECs were grown in DMEM/F12, supplemented with 5% FBS, 2 mM glutamine (Sigma-Aldrich) and antibiotics (Sigma-Aldrich), grown in 5% CO_2_ at 37°C, and passaged once a week at 1:3 ratio.

#### 2.2.2 Endometrial 3D culture model assembly

12-well Alvetex^®^ Scaffold inserts (Reprocell Europe, United Kingdom) were prepared according to manufacturer’s instructions. 1 × 10^6^ eSFs were seeded onto inserts at days 0, 7 and 9 of culture and grown in DMEM supplemented with 10% FBS, 2 mM glutamine (Sigma-Aldrich), 100 μg/mL ascorbic acid (Sigma-Aldrich), and antibiotics (Sigma-Aldrich) in 5% CO_2_ at 37°C for 14 days. Medium was refreshed twice a week.

On day 14, 2 × 10^6^ eECs were layered on the stromal compartment and maintained in DMEM/F12 supplemented with 5% FBS, 2 mM glutamine (Sigma-Aldrich) and antibiotics (Sigma-Aldrich) for 21 days. Cultures were maintained in 5% CO_2_ at 37°C and medium was refreshed twice a week.

All experiments were performed at least three times in triplicates.

### 2.3 Generation of trophoblast spheroids

#### 2.3.1 Dermal fibroblast isolation, growth, and maintenance onto standard plastic dishes

Five porcine abdominal skin biopsies were cut in small fragments of approximately 2 mm^3^, transferred onto 0.1% gelatin (Sigma-Aldrich) pre-coated Petri dishes (Sarstedt) and cultured in DMEM supplemented with 20% FBS, 2 mM glutamine (Sigma-Aldrich) and antibiotics (Sigma-Aldrich). After 6 days of culture, fibroblasts started to grow out of the explant fragments and the latter were carefully removed. Fibroblasts were maintained in the medium described above, grown in 5% CO_2_ at 37°C and passaged twice a week in a 1:3 ratio.

#### 2.3.2 Treatment of dermal fibroblasts with 5-aza-CR and trophoblast induction into micro-bioreactors

Dermal fibroblasts were epigenetically erased with 1 μM 5-aza-CR (Sigma-Aldrich) for 18 h and encapsulated into polytetrafluoroethylene (PTFE) micro-bioreactors. Concentration and time of exposure were selected according to our previous works (Pennarossa et al., 2019; 2023a; 2024). Specifically, PTFE powder bed with particle size of 1 μm (Sigma-Aldrich, #430935) was created inside a Petri dish and 4 × 10^4^ cells resuspended in 30 μL of 1 μM 5-aza-CR were dispensed on it. Petri dish was gently shaken in a circular motion to ensure that PTFE powder particles completely covered the surface of the liquid drop, creating a micro-bioreactor that was incubated at 37°C in 5% CO_2_ in air. At the end of the 5-aza-CR exposure, micro-bioreactors were broken by puncturing with a needle. Formed spheroids were recovered using a 200 μL pipette tip cut at the edge, re-encapsulated into new PTFE micro-bioreactors and cultured in embryonic stem cell (ESC) medium consisting of DMEM-low glucose:HAM’S F10 (1:1) supplemented with 5% FBS, 10% K.O. serum, 2 mM glutamine, 0.1 mM β-mercaptoethanol, 1% nucleoside mix (guanosine 0.84 g/L, adenosine 0.8 g/L, cytidine 0.72 g/L, uridine 0.72 g/L, and timidine 0.24 g/L), 1% non-essential amino acids, 1000IU/mL ES-growth (LIF, Chemicon), 5 ng/mL b-FGF (R&D System) (Brevini et al., 2010) for 3 h at 37°C in 5% CO_2_. Subsequently, trophectoderm differentiation was induced by culturing formed spheroids into PTFE micro-bioreactors and using mouse embryonic fibroblast (MEF) conditioned medium (Arcuri et al., 2021a; 2021b) supplemented with 10 ng/mL bone morphogenetic protein 4 (BMP4, Sigma-Aldrich), 1 µM activin/nodal signaling inhibitor (A83-01, Sigma-Aldrich) and 0.1 µM FGF2 signaling inhibitor (PD173074, Sigma-Aldrich) (Amita et al., 2013). Culture was carried out at 37°C in low O_2_ condition (5% O_2_, 5% CO_2,_ and 90% N_2_ atmosphere) for 11 days and culture medium was refreshed every other day. To increase humidity and avoid dehydration, the Petri dishes containing micro-bioreactors were placed in a larger dish containing sterile water.

### 2.4 Development of mammalian implantation *in vitro* model

At the end of 11-day culture period, TR spheroids were transferred onto the top of the generated 3D endometrial models and maintained in culture for 24 h in DMEM/F12 supplemented with 5% FBS, 2 mM glutamine (Sigma-Aldrich) and antibiotics (Sigma-Aldrich). Samples were then fixed and subjected to histological evaluation.

### 2.5 Gene expression analysis

RNA was extracted from 3D models using the RNeasy mini kit (Qiagen) following the Reprocell’s instructions (https://www.reprocell.com/protocols/alvetex-scaffold/d045-rna-extraction-from-cells). DNase I (1:100) was added in lysis solution. Quantitative Real-Time PCR was carried out using predesigned gene-specific primers and probe sets (TaqManGene Expression Assays, Table 1). GAPDH and ACTB genes were selected as reference genes. Target gene analysis was performed using CFX96 Real-Time PCR detection system (Bio-Rad Laboratories) and CFX Manager software (Bio-Rad Laboratories). Porcine endometrial tissue was used as a positive control.

**TABLE 1:** List of primers used for quantitative PCR analysis.

Gene	Description	Catalog no.
ACTB	Actin beta	Ss03376563_uH
COL3A1	Collagen Type III Alpha 1 Chain	Ss04323794_m1
CDH1	Cadherin 1	Ss06942341_m1
KRT18	Keratin 18	Ss03377383_u1
GAPDH	Glyceraldehyde-3-phosphate dehydrogenase	Ss03375629_u1
EPCAM	Epithelial Cell Adhesion Molecule	Ss03384752_u1
GCM1	Glial cells missing transcription factor 1	Ss03373780_m1
HSD17B1	Hydroxysteroid 17-beta dehydrogenase 1	Ss04245960_g1
IFNG	Interferon gamma	Ss03391054_m1
NANOG	Nanog homeobox	Ss04245375_s1
OCT4	POU class 5 homeobox 1	Ss03389800_m1
COX1	Prostaglandin-Endoperoxide Synthase 1	Ss03373347_m1
COX2	Prostaglandin-Endoperoxide Synthase 2	Ss03394694_m1
PAG6	Pregnancy-associated glycoprotein 6	Ss03378057_u1
PPAG3	Pregnancy-associated glycoprotein 3	Ss03392369_m1
REX1	ZFP42 zinc finger protein	Ss03373622_g1
PGFS	Prostaglandin F Synthase	Ss03387233_u1
PTGES	Prostaglandin E Synthase	Ss03392129_m1
SOX2	Sex determining region Y-box 2	Ss03388002_u1
THY1	Thy-1 cell surface antigen	Ss03376963_u1
VIM	Vimentin	Ss04330801_gH
ZO1	Tight Junction Protein ZO-1	Ss03373514_m1

### 2.6 Histological evaluations

At the end of culture, 3D models were fixed in 4% PFA for 24 h, dehydrated through a series of ethanol, incubated in Histoclear (Bio-optica) and embedded in paraffin. 5–7 µm thick sections were cut, dewaxed, re-hydrated, and stained with hematoxylin/eosin (HE) or with 4′,6-diamidino-2-phenylindole (DAPI). Samples were analyzed using Leica DMR microscope (Leica Microsystems).

### 2.7 Immunocytochemical and immunohistochemical analysis

Immunocytochemical analysis was used to identify and localize the TR marker GATA Binding Protein 3 (GATA3) in the generated TR spheroids. Samples were fixed in 4% paraformaldehyde for 20 min, washed three times in PBS, permeabilized with 0.5% Triton X-100 (Sigma) for 30 min, and treated with a blocking solution containing 10% goat serum (Sigma) for 30 min. An overnight incubation with primary anti-GATA3 monoclonal antibody (# 14-9966-82, 1:250) diluted in PBS was carried out at + 4°C. The day after, samples were washed three times in PBS and incubated with Alexa FluorTM 594 goat anti-rat secondary antibody for 45 min at room temperature, using a 1:250 dilution. Nuclei were stained with 4′,6-diamidino-2-phenylindole (DAPI). At the end of the immunostaining procedure, TR spheorids were analyzed under an Eclipse E600 microscope (Nikon) equipped with a digital camera (Nikon).

Immunohistochemistry analyses on paraffin-embedded sections were used to detect specific targets. In particular, stromal compartment was characterized through immunohistochemical detection of vimentin (VIM), epithelial layer was identified through tight junction formation and the expression of zonula occludens 1 (ZO1), and trophoblast spheroid attachment to the generated 3D endometrial model was analyzed through the detection of GATA3 and ZO1. Briefly, slides were brought to boiling in 10 mM sodium citrate buffer, 0.05% Tween-20 (pH 6) in a pressure cooker for 1 min for antigen retrieval. No-specific binding was prevented by incubating sections in 10% bovine serum albumin (BSA) for 30 min at room temperature. Samples were then incubated with anti-vimentin antibody (Abcam, # ab8978, 1:250) and FITC-conjugated anti-Zo-1 antibody (#33-9100, 1:100), or with anti-GATA3 monoclonal antibody (# 14-9966-82, 1:250) diluted in PBS for 1 h at room temperature. Subsequently, sections were exposed to the adequate Alexa FluorTM 594 goat anti-mouse secondary antibody (for VIM) and Alexa FluorTM 594 goat anti-rat secondary antibody (for GATA3) diluted 1:250 in PBS for 30 min at room temperature. Nuclei were counterstained with DAPI. Negative controls were performed following the same staining protocol but omitting the primary antibody.

### 2.8 Functional assessment

#### 2.8.1 Measurement of transepithelial electrical resistance (TEER)

Endometrial barrier TEER was measured using an EVOM2 Epithelial Voltmeter with STX3 electrode (World Precision Instrument). The electrode was equilibrated according to the manufacturer’s instruction. The electrode was insert in the 3 sides of each insert and the final TEER value was determined as follows:

TEER (Ohm x cm^2^) = (TEER average sample insert–TEER average blank insert) x Area cm^2^.

#### 2.8.2 Prostaglandin E2 release

Endometrial 3D models were treated for 24 h with 100 nm oxytocin and 100 μm arachidonic acid as previously described (MacKintosh et al., 2015; Díez et al., 2023). Treatment was applied to both apical and basolateral compartments and supernatants were collected and analyzed separately using the Prostaglandin E2 ELISA Kit (Abcam, #ab287802) and following the manufacturer’s instructions. Each sample was assayed in triplicate and at the end of the procedures, absorbances were measured at 450 nm. Standard curves were constructed by plotting the mean absorbance (*y*-axis) against PGE2 concentration (*x*-axis) and the best fit line was determined by regression analyses. Absolute quantifications were then calculated.

### 2.9 Statistical analysis

Statistical analysis was performed using two-way ANOVA (SPSS 19.1; IBM). Data were presented as mean ± standard deviation (SD). Differences of *p* ≤ 0.05 were considered significant and were indicated with different superscripts.

## 3 Results

### 3.1 Creation and functional evaluation of the 3D endometrial model

**FIGURE 1:**
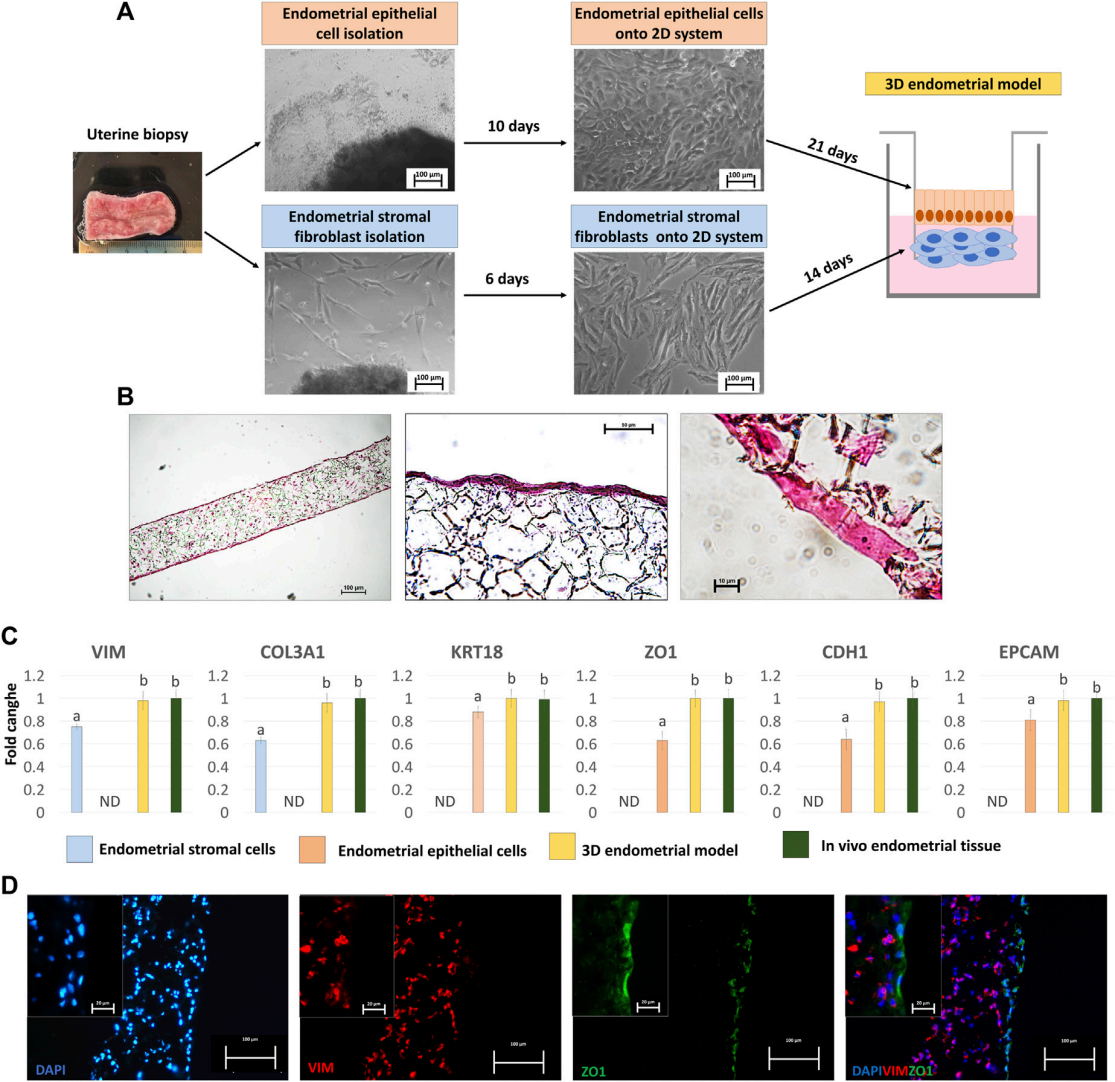
Generation of the porcine 3D endometrial model and its characterization. **(A)** Schematic representation of the 3D endometrial model assembling, from uterine biopsy to endometrial epithelial cell and stromal fibroblast isolation and culture onto 2D standard plastic dishes (scale bar 100 μm). **(B)** Hematoxylin and Eosin staining of the 3D endometrial model obtained by co-culturing epithelial cells and stromal fibroblasts onto highly porous polystyrene scaffolds (scale bars 100 and 50 μm). **(C)** Transcription levels of VIM, COL3A1, KRT18, ZO1, CDH1 and EPCAM genes in endometrial stromal cells cultured on 2D culture systems (blue bars), endometrial epithelial cells cultured on 2D culture systems (pink bar), 3D endometrial models (yellow bars) and *in vivo* endometrial tissue, as a positive control (green bars). Gene expression is presented with the highest level set to 1 and all others relative to this. Data are expressed as the mean ± the standard error of the mean (SEM). ^a,b^Different superscripts indicate *p* < 0.05. ND: not detected. **(D)** Immunofluorescent staining of 3D endometrial models for VIM (red) and ZO1 (green). Nuclei are counterstained with DAPI (blue) (scale bars 100 and 20 μm).

Endometrial stromal fibroblasts (eSFs) and epithelial cells (eECs) grew out of the original explants within 6 and 10 days of culture, respectively (Figure 1A). Both cell types formed a monolayer and displayed the appropriate standard morphology, fusiform bodies with oval nuclei for eSFs (Figure 1A, lower panel) and polygonal shape with centrally placed round nuclei for eECs (Figure 1A, upper panel).

When engrafted onto highly porous polystyrene scaffolds, eSFs formed a 3D stromal layer characterized by a highly dense cellular stratum, starting from 14 days of culture onward (Figure 1B), thus ensuring the creation of a robust structural support that mimics the subepithelial compartment. eECs were then plated onto the generated 3D stromal structure and cultured for further 21 days. Histological characterization, carried out at the end of the culture period, showed cells with a distinct polarized morphology and a more elongated columnar shape (Figure 1B), distinctive of epithelial cells in *in vivo* tissues.

Molecular analysis confirmed the acquisition of a mature phenotype with significant upregulation of the main stromal and epithelial markers, namely, VIM, COL3A1, KRT18, ZO1, CDH1and EPCAM, in cells grown onto scaffolds compared to those cultured in 2D monolayers (Figure 1C). Interestingly, the values detected in the generated 3D endometrial models were statistically comparable to *in vivo* endometrial tissue used as a positive control (Figure 1C). These results were also paralleled by VIM and ZO1 immunopositivity of stromal and epithelial compartments, respectively (Figure 1D).

**FIGURE 2:**
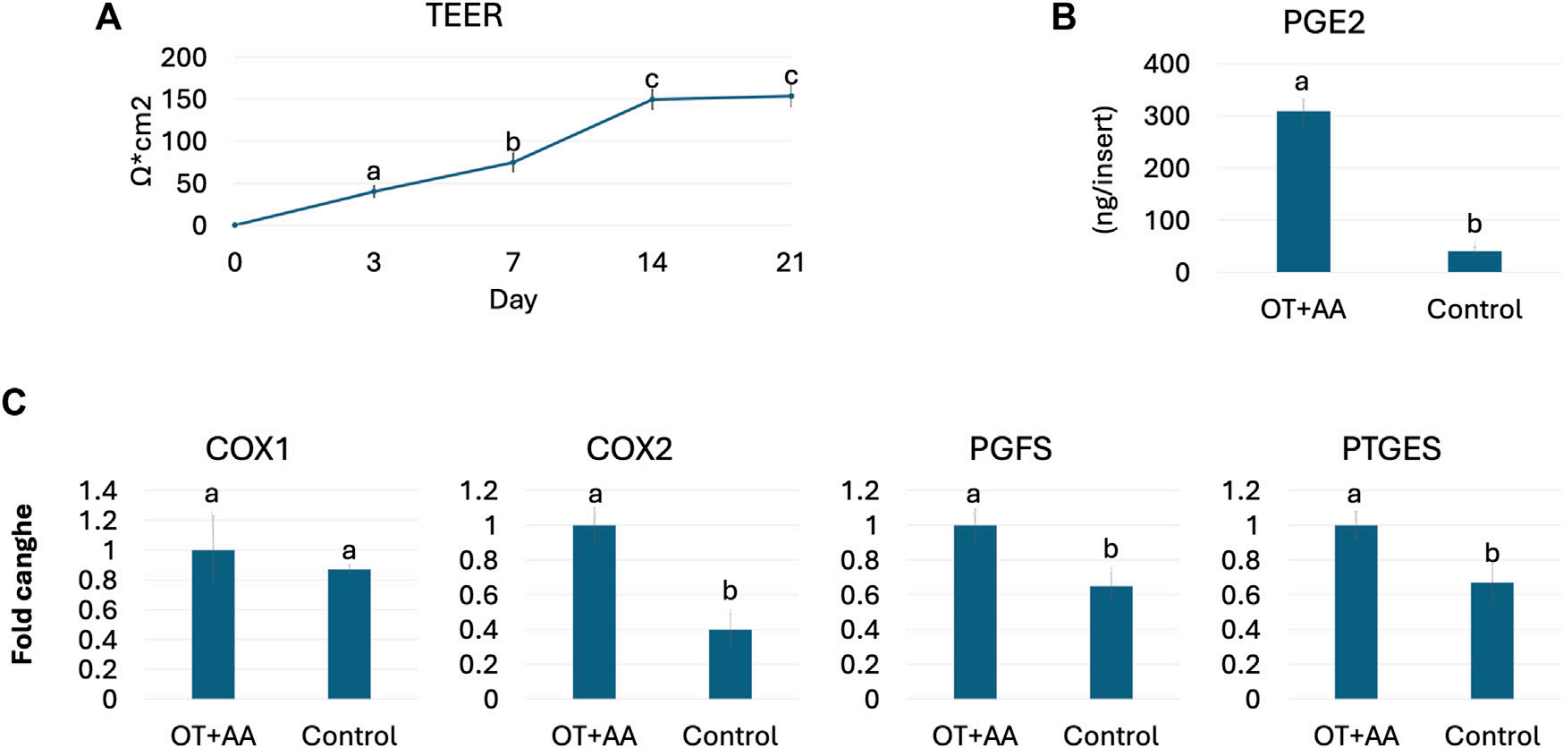
Porcine 3D endometrial model validation. **(A)** TEER values detected in 3D endometrial model obtained by co-culturing endometrial stromal and epithelial cells onto highly porous polystyrene scaffolds at different time points. Data are expressed as the mean ± the standard error of the mean (SEM). ^a,b,c^Different superscripts indicate *p* < 0.05. **(B)** PGE2 release in 3D endometrial models after 24-h OT + AA stimulation (OT + AA) and in untreated cells (Control). Data are expressed as the mean ± the standard error of the mean (SEM). ^a,b^Different superscripts indicate *p* < 0.05. **(C)** Transcription levels of COX1, COX2, PGFS and PTGES genes in 3D endometrial models after 24-h OT + AA stimulation (OT + AA) and in untreated cells (Control). Gene expression is presented with the highest value set to 1 and all others relative to this. Data are expressed as the mean ± the standard error of the mean (SEM). ^a,b^Different superscripts indicate *p* < 0.05.

Functional studies demonstrated an average TEER value of 153.66 ± 12.54 Ω*cm^2^ at the end of the culture period (Figure 2A). In addition, OT and AA treatment induced an increased PGE2 release (OT + AA, Figure 2B), compared to untreated 3D endometrial models (Control, Figure 2B). This increment was also paralleled by higher expression levels for COX1, COX2, PGFS, and PTGEs genes, when compared to the untreated control group (Figure 2C).

### 3.2 Generation and characterization of TR spheroids

**FIGURE 3:**
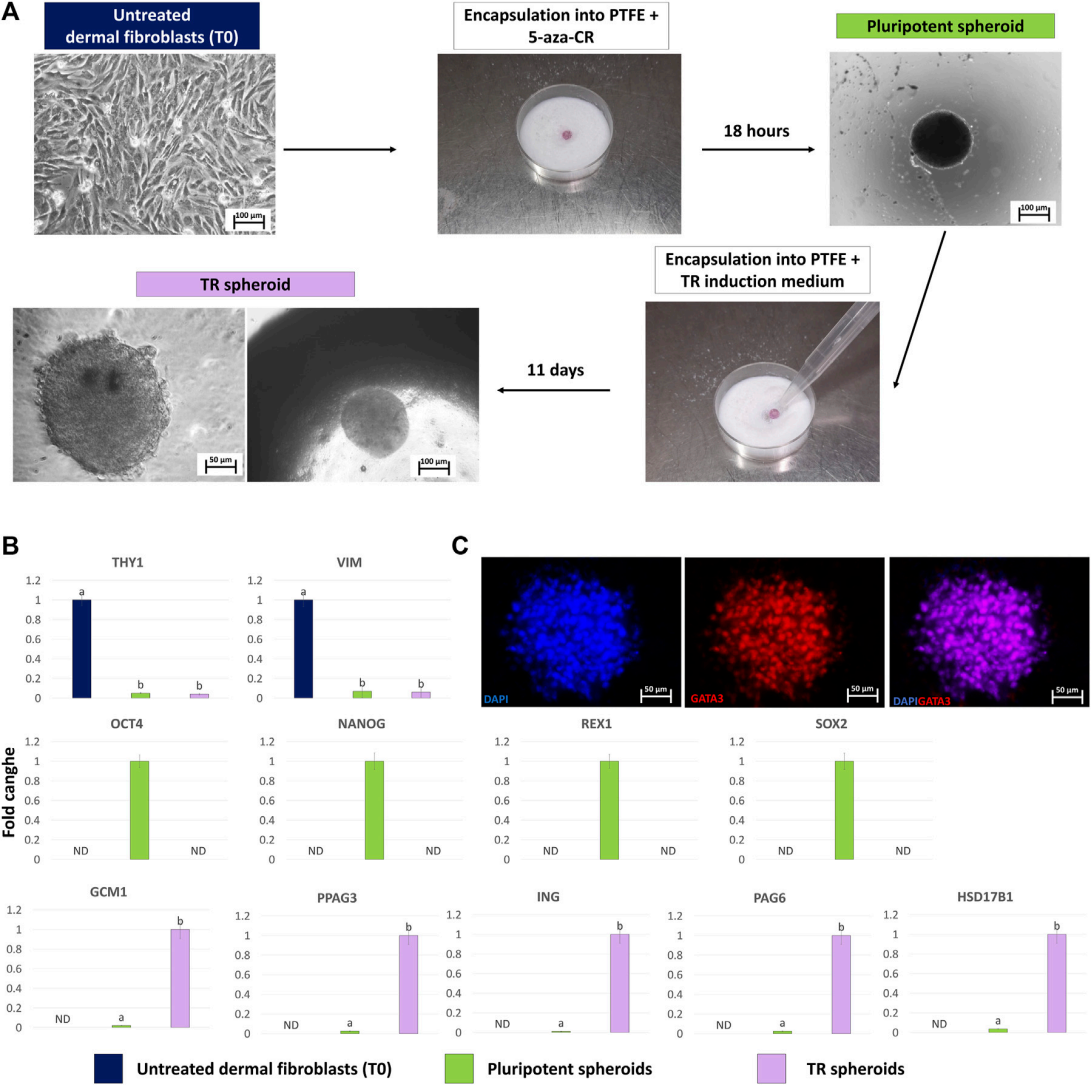
Generation of TR spheroids and their characterization. **(A)** Dermal fibroblasts grown into 2D culture systems were resuspended in medium containing 1uM 5-aza-CR and encapsulated onto PTFE micro-bioreactor. After 18 h of culture, the obtained pluripotent spheroid was recovered, encapsulated into a new micro-bioreactor and cultured for 11 days using a TR induction medium (scale bars 100 and 50 μm). **(B)** Transcription levels for fibroblast- (THY1 and VIM), pluripotent- (OCT4, NANOG, REX1, and SOX2) and TR-related (GCM1, PPAG3, ING, PAG6, HSD17B1) genes in untreated fibroblasts (T0, blue bars), in pluripotent spheroids (green bars) and in TR spheroids (lilac bars).Gene expression is represented with the highest value set to 1 and all others relative to this. Data are expressed as the mean ± the standard error of the mean (SEM). ^a,b^Different superscripts indicate *p* < 0.05. ND: not detected. **(C)** Representative images of a TR spheroid immunostained for GATA3. Nuclei are counterstained with DAPI (blue) (scale bars 50 μm).

Fibroblasts grew out of the skin biopsy fragments within 6 days of culture, formed a monolayer, and displayed the standard elongated morphology (Figure 3A, Untreated dermal fibroblasts, T0). After encapsulation into PTFE micro-bioreactors and exposure to 5-aza-CR, cells became rounded, with large and granulated nuclei and rearranged into 3D spherical structures (Figure 3A, Pluripotent spheroid). These morphological changes were accompanied by the onset of the pluripotency-related genes, OCT4, NANOG, REX1, and SOX2, which were not transcribed by untreated fibroblasts (T0, Figure 3B). In contrast, transcription levels of the fibroblast specific genes, THY1 and VIM, significantly decreased after encapsulation and 5-aza-CR treatment (Figure 3B). Subsequent induction with TR medium carried out into PTFE micro-bioreactors drove cells towards TR lineage and, at the end of culture period, cells showed a mature TR morphology, exhibiting round or ellipsoid shape, round nuclei, and well-defined borders, while maintaining a 3D spherical organization (Figure 3A, TR spheroid). This was paralleled by an active expression of the mature TR markers GCM1, PPAG3, IFNG, PAG6, and HSD17B1, which were undetectable in untreated fibroblasts (Figure 3B, T0) as well as by TR spheroid immunopositivity for GATA3 (Figure 3C).

### 3.3 Development of the mammalian implantation *in vitro* model

**FIGURE 4:**
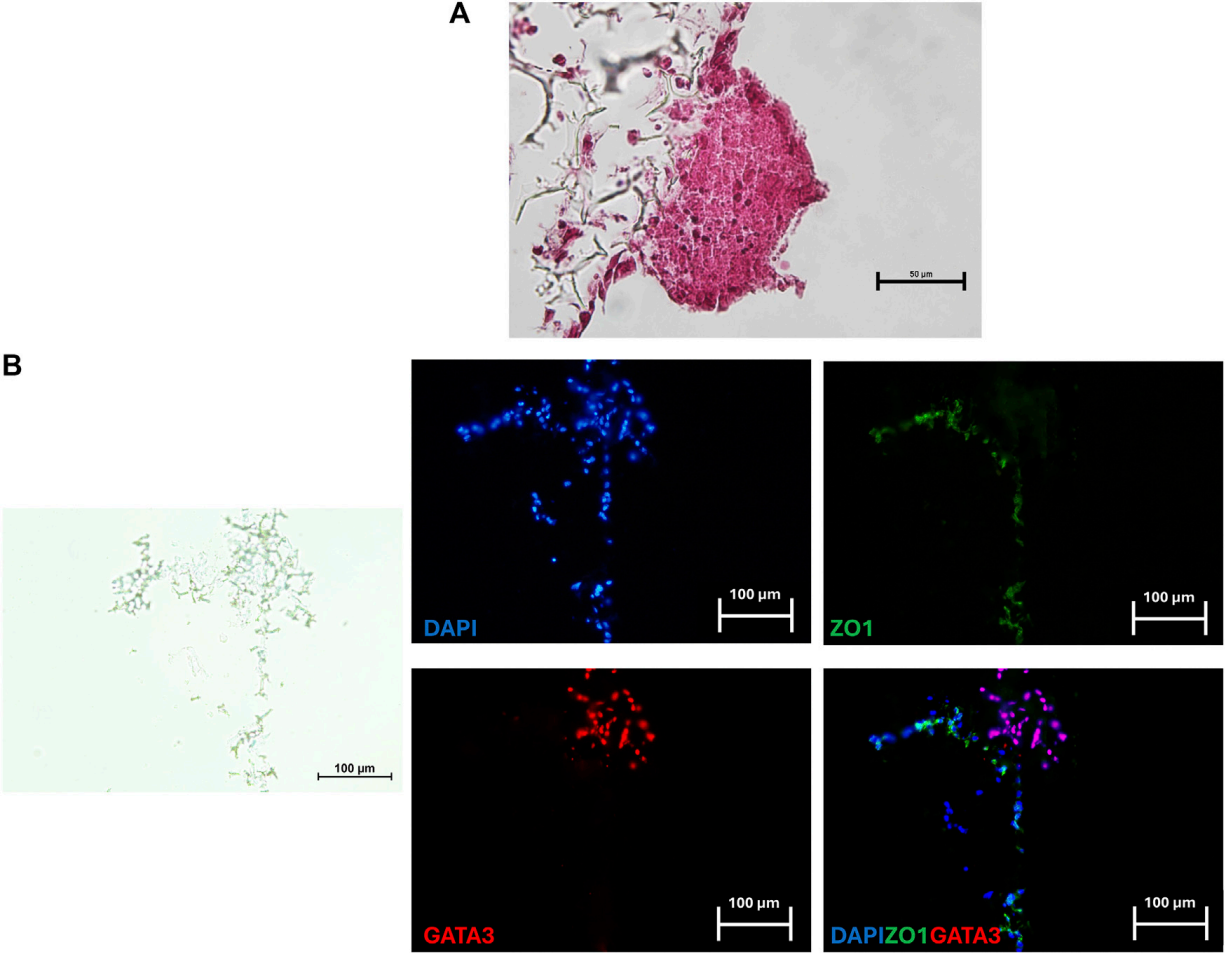
Representative image of a TR spheroid attached to the 3D endometrial model. **(A)** Hematoxylin and Eosin staining of a TR spheroid adhering to the 3D endometrial culture system after a 24-h co-culture. No space between the spheroid and the epithelial compartment was visible (scale bar 50 μm). **(B)** Immunohistochemical co-staining for the mature TR marker GATA3 (red) and ZO1 (green) of a TR spheroid adhering to the 3D endometrial culture system. Nuclei were counterstained with DAPI (blue) (scale bars 100 μm).

After a 24-h co-culture, TR spheroids adhered to the generated 3D artificial endometrium. H&E staining showed spheroids closely attached to the 3D endometrial platform. No space between spheroids and epithelial cells was visible, and, in contrast, TR spheroids seemed to displace the epithelial compartment on top of the culture system (Figure 4A). GATA3/ZO1 immunohistochemical co-staining indicated TR spheroid attachment to the artificial endometrium that, as expected, was negative for GATA3 and positive for ZO1 (Figure 4B).

## 4 Discussion

In the present work, we describe, for the first time, the development of a reproducible and functional implantation *in vitro* model in the porcine species. We first separately generate an artificial endometrium and TR spheroids. We then combine the two, to simulate *in vitro* the interactions occurring between the maternal and the embryo compartments.

In the first set of experiments, we created the maternal tissue starting from porcine freshly isolated eSFs, which were plated onto highly porous polystyrene scaffolds. After a triple seeding and 14 days of culture, eSFs were able to successfully repopulate the scaffold inserts forming a robust structural support that closely resembles the subepithelial compartment. As demonstrated by the histological staining, fibroblasts preserved their native shape and display the typical tissue 3D organization. These observations suggest that the 3D culture system used properly promote cell-to-cell interactions, as well as an *in vivo*-like spatial arrangement. In addition, consistent with previous studies performed using the same scaffold in combination with primary dermal or intestinal fibroblasts (Darling et al., 2020; Costello et al., 2021; Pennarossa et al., 2023b; Díez et al., 2023), cells colonized the whole culture support thickness, thus demonstrating that the highly porous polystyrene scaffold is able to support cell adhesion and growth.

eECs subsequently plated onto the generated 3D stromal compartment, and grown for further 21 days, showed a monolayer organization, losing the flat morphology -distinctive of 2D systems- and acquired a cubic/cylindric shape, typical of polarized epithelial cells *in vivo*. This is in agreement with what recently reported by Díez et al. (2023), who generated an artificial bovine endometrial model *in vitro*. It is also in line with Mitra et al.,(2018), Darling et al. (2020), and Pennarossa et al. (2023b) who observed a distinct polarization of intestinal epithelial cells obtained from different species, ranging from the human to the porcine, when seeded on the same culture support here described. In addition, the stromal compartment was immunopositive for VIM, while the epithelial one for ZO1. This indicates the presence of tight junctions that, *in vivo*, interacts with other proteins belonging to the classes of cadherin and claudin, contributing to the maintenance of a functional epithelial barrier and to signal transduction, thus suggesting the formation of a tightly aligned epithelium and a functional barrier. Interestingly, morphological observations are paralleled by the molecular results that indicate a significant upregulation of the main stromal and epithelial markers, namely, VIM, COL3A1, KRT18, ZO1, CDH1and EPCAM, in cells grown onto highly porous polystyrene scaffolds compared to those cultured onto standard 2D dishes, further confirming cell ability to acquire a mature phenotype. Moreover, barrier integrity assessment, at the end of the culture period, demonstrated an average TEER value of 153.66 ± 12.54 Ω*cm^2^. Unfortunately, no data on endometrium electrical resistance *in vivo* are, to our knowledge, currently available in the literature, making difficult a physiological interpretation of our results. Nevertheless, our data are consistent with previous *in vitro* studies performed with human and monkey oviductal epithelial cells cultured onto 3D models, that showed similar TEER values (Mahmood et al., 2001; Rajagopal et al., 2006), suggesting the successful generation of a functional organotypic epithelial barrier with good integrity. Functionality of the newly generate endometrial platform was further evaluated assessing its ability to release PGE2 in response to OT and AA stimulation. The results obtained demonstrate a significant increase in the prostaglandin concentration measured in 3D endometrial model supernatants compared to those obtained from untreated ones, well-fitting with previous reports demonstrating PGE2 increment in bovine polarized epithelial cells co-cultured with stromal cells (MacKintosh et al., 2015; Díez et al., 2023). In addition, our experiments show that PGE2 increment is also paralleled by upregulated transcriptions of COX1, COX2, PGFS, and PTGES genes, further confirming an active and orchestrated activation of the prostaglandin pathway in the generated artificial endometrium.

Encapsulation into PTFE micro-bioreactors and exposure to 5-aza-CR of porcine adult dermal fibroblasts coaxed cells into 3D spherical structures. This is consistent with previous studies describing PTFE ability to encourage cell aggregation and allow cell to self-assemble forming multicellular spheroids, with uniform size geometry (Sarvi et al., 2013; Vadivelu et al., 2017; Pennarossa et al., 2019; Sneha Ravi and Dalvi, 2024). In addition, cells responded to chemical reprogramming displaying rounded morphology, with large and granulated nuclei, and actively expressing the pluripotency-related genes, OCT4, NANOG, REX1, and SOX2, which were not transcribed by untreated fibroblasts. These morphological and molecular changes are in agreement with the 5-aza-CR well-known ability to reactivate silent genes (Taylor and Jones, 1979; Jones, 1985; Glover et al., 1986) and to induce a high plasticity state in adult somatic cells (Palii et al., 2008; Mirakhori et al., 2015; Chandrakanthan et al., 2016; Diomede et al., 2018; Pennarossa et al., 2019), clearly indicating the acquisition of a specific pluripotent phenotype. Taking advantage of the acquired high permissivity state, cells were readdressed toward the TR lineage using an induction medium containing BMP4 and inhibitors of the activin/nodal and FGF2 signaling pathways (Sudheer et al., 2012; Amita et al., 2013; Lee et al., 2015; Yang et al., 2015; Yabe et al., 2016; Jain et al., 2017; Koel et al., 2017; Roberts et al., 2018; Sheridan et al., 2019; Karvas et al., 2020). The acquisition of a TR-like phenotype at the end of the 11-day culture period, further support previous reports demonstrating the key role of FGF2 and activin-a/nodal signaling suppressors to drive cells along a complete and unidirectional differentiation pathway towards the TR lineage (Schulz et al., 2009; Ezashi et al., 2012; Amita et al., 2013; Roberts et al., 2018). The mature TR morphology observed in our experiments is also consistent with Assheton (1906) and Hou et al. (2015) that described round or ellipsoid shape, round nuclei, and well-defined borders, in porcine placental *in vivo* tissues and in TR cells isolated from *in vitro*-produced porcine blastocysts and parthenotes, respectively. Progression toward the new TR phenotype is also confirmed by an active expression of the mature TR markers GCM1, PPAG3, IFNG, PAG6, HSD17B1, which were undetectable in untreated fibroblasts, as well as by GATA3 immunopositivity of TR spheroids, detected at the end of the 11-day lineage specific induction.

Interestingly, when TR spheroids were co-cultured with the generated artificial endometrium for 24 h, they closely adhered to the 3D maternal model, with no visible space between the two compartments and displacement of the endometrial epithelial cells. This is coherent with Wang et al. (2012) who described higher attachment rates between Jar cell trophoblastoids and complete Ishikawa/T HESC-matrix 3D structures, compared to the same trophoblastoid and 3D systems consisting of only Ishikawa cells and matrix, without any stromal cells. Based on all these observations, we may hypothesize that stromal/epithelial cell interactions may exert a strong influence on the trophoblast compartment, promoting its attachment.

Altogether, the results obtained in the present study demonstrate the possibility to generate reproducible and functional 3D models of both maternal and embryo compartments that can find useful applications for a better understanding of early implantation mechanisms. In addition, the two 3D culture systems here described are advantageous in order to mimic *in vitro* the complex architecture and physiology of their native counterparts *in vivo* and represent useful bio-engineered tools to study the complex interactions between the maternal tissue and the developing embryos.

## Data Availability

The original contributions presented in the study are included in the article/Supplementary Material, further inquiries can be directed to the corresponding author.
